# Management of acute urinary retention: a worldwide survey of 6074 men with benign prostatic hyperplasia

**DOI:** 10.1111/j.1464-410X.2011.10430.x

**Published:** 2012-01

**Authors:** John M Fitzpatrick, François Desgrandchamps, Kamel Adjali, Lauro Gomez Guerra, Sung Joon Hong, Salman El Khalid, Krisada Ratana-Olarn

**Affiliations:** Department of Urology, Mater Misericordiae University Hospital and University CollegeDublin, Ireland; * Department of Urology, Saint-Louis HospitalParis, France; † Department of Urology, Bab El Oued University HospitalAlger, Algeria; ‡ Department of Urology, University HospitalNuevo Leon, Mexico; § Yonsei University College of MedicineSeoul, Korea; ¶ The Kidney Centre InstituteKarachi, Pakistan; ** Mahidol UniversityBangkok, Thailand

**Keywords:** acute urinary retention, alfuzosin, α_1_-blocker, benign prostatic hyperplasia, trial without catheter

## Abstract

**OBJECTIVES:**

- To evaluate the management of acute urinary retention (AUR) associated with benign prostatic hyperplasia (BPH) in real-life practice.
- To identify predictors of successful trial without catheter (TWOC).

**MATERIALS AND METHODS:**

- In all, 6074 men catheterized for painful AUR were enrolled in a prospective, cross-sectional survey conducted in public and private urology practices in France, Asia, Latin America, Algeria and the Middle East.
- Patient clinical characteristics, type of AUR and its management (type of catheterization, hospitalization, TWOC, use of α_1_-blockers, immediate or elective surgery) and adverse events observed during the catheterization period were recorded.
- Predictors of TWOC success were also analysed by multivariate regression analysis with stepwise procedure.

**RESULTS:**

- Of the 6074 men, 4289 (71%) had a spontaneous AUR and 1785 (29%) had a precipitated AUR, mainly as the result of loco-regional/general anaesthesia (28.5%) and excessive alcohol intake (18.2%).
- Presence of BPH was revealed by AUR in 44% of men. Hospitalization for AUR varied between countries, ranging from 1.7% in Algeria to 100% in France. A urethral catheter was inserted in most cases (89.8%) usually followed by a TWOC (78.0%) after a median of 5 days. Overall TWOC success rate was 61%.
- Most men (86%) received an α_1_-blocker (mainly alfuzosin) before catheter removal with consistently higher TWOC success rates, regardless of age and type of AUR. Multivariate regression analysis confirmed that α_1_-blocker before TWOC doubled the chances of success (odds ratio 1.92, 95% CI 1.52–2.42, *P* < 0.001).
- Age ≥70 years, prostate size ≥50 g, severe lower urinary tract symptoms, drained volume at catheterization ≥1000 mL and spontaneous AUR favoured TWOC failure. Catheterization >3 days did not influence TWOC success but was associated with increased morbidity and prolonged hospitalization for adverse events.
- In the case of TWOC failure, 49% of men were recatheterized and had BPH surgery and 43.5% tried another TWOC with a success rate of 29.5%. Elective surgery was preferred to immediate surgery.

**CONCLUSIONS:**

- TWOC has become a standard practice worldwide for men with BPH and AUR.
- In most cases, an α_1_-blocker is prescribed before TWOC and significantly increases the chance of success.
- Prolonged catheterization is associated with an increased morbidity.

**What's known on the subject? and What does the study add?**

Largest survey ever conducted evaluating the management of AUR in real life practice in a wide range of health care systems. It shows that urethral catheterization followed by a TWOC has become a standard worldwide and that α_1_-blockade prior to TWOC doubles the chances of success. It also evidences important differences (hospitalization rate, duration of catheterization …) between countries/regions reflecting lack of guidelines. This large survey also clearly identifies predictors of TWOC failure.

## INTRODUCTION

Acute urinary retention (AUR) is a severe complication of BPH characterized by a sudden and painful inability to void voluntarily [1]. It is a distressing condition [2] that represents a major public health issue. Within the last decade, AUR has become the most common indication for TURP, increasing from 22.9% in 1988 to 42.9% in 1998 [3]. In addition, a recent analysis of 176 046 men admitted to NHS hospitals in England for AUR between 1998 and 2005 has shown that mortality within the year after a first AUR episode was much higher than in the general population, especially in younger patients [4].

In clinical practice, AUR is usually attributed to BPH, as being part of its natural history (so-called ‘spontaneous AUR’). Data from large community-based longitudinal studies have identified old age, severe lower urinary tract symptoms (LUTS), low peak flow rate, high postvoid residual urine (PVR), enlarged prostate and high serum PSA as significant risk factors for spontaneous AUR [5,6]. Dynamic variables such as symptom worsening and increasing PVR during medical therapy are also powerful predictors of AUR [7,8]. In other cases, AUR develops after a precipitating event other than BPH (so-called ‘precipitated’ AUR), for example a surgical procedure with general or loco-regional anaesthesia, bladder overdistension, urinary tract infection, medications with sympathomimetic or anticholinergic effects [9]. The differentiation between both types of AUR is relevant in clinical practice because precipitated AUR is associated with a much higher mortality rate at 1 year (24-fold increase vs general population) than spontaneous AUR (10-fold increase) [4]. This is possibly explained by a higher prevalence of severe underlying comorbid conditions in men with precipitated AUR and this may have important implications in terms of AUR management.

Management of AUR consists of immediate bladder decompression by catheterization usually followed by BPH-related surgery. The evidence that emergency surgery was associated with an increased mortality rate at 30 days and a higher rate of postoperative complications, and the potential morbidity associated with prolonged catheterization have led to an increasing use of a trial without catheter (TWOC) [9,10]. A TWOC involves catheter removal, usually after 2–3 days of α_1_-blockade, allowing the patient to return to normal voiding in up to 60% of cases [10,11]. The benefit of α_1_-blockade on the TWOC success rate was shown in a large double-blind, placebo-controlled study that randomized 360 men with AUR to alfuzosin 10 mg once daily or placebo for 2–3 days followed by a TWOC [11]. It was further established by a recent Cochrane metaanalysis [12]. This TWOC policy is likely to have contributed to the decrease in the number of TURPs after a first AUR episode in the UK but balanced against this is a slight increase in the AUR recurrence rate [13].

Nevertheless, there is currently no consensus on the optimal management of AUR, in terms of type of catheterization, duration of catheterization and management after catheterization. The Reten-World survey aimed to evaluate prospectively the current practice in management of AUR associated with BPH, in various regions (France, Latin America, Asia, Algeria, Middle East) representing a wide range of healthcare systems.

## METHODS

In all, 6074 men presenting with a painful AUR related to BPH were enrolled between April 2004 and April 2008 by 953 urologists from public (37%), private (42%) or mixed (21%) healthcare practices in a prospective cross-sectional survey conducted in France (*n* = 2618), Asia (Korea, Pakistan, Philippines, Taiwan, Thailand, Vietnam; *n* = 1727), Latin America (Colombia, Mexico, Venezuela; *n* = 883), Algeria (*n* = 755) and the Middle East (Bahrain, Qatar, Kuwait, United Arab Emirates; *n* = 91). The results of the French survey have already been published [14]. Patient demographic data, history of BPH, prostate size estimated by DRE, type of AUR (spontaneous or precipitated), amount of drained volume, management of AUR (type and duration of catheterization, TWOC, use of α_1_-blockers before TWOC, hospitalization rate, immediate or elective surgery) and adverse events observed during the catheterization period were recorded. Written informed consent was obtained from all patients. Study protocol was approved by the ethics committees and Health Authorities, according to local legislation.

Statistical comparisons were made using chi-squared or Fisher exact test for qualitative variables and Student's *t* test for quantitative variables. A *P* value < 0.05 was considered statistically significant. A multiple logistic regression analysis with stepwise procedure was performed to determine whether the TWOC success rate was influenced by selected variables (age <70 or ≥70 years, LUTS severity according to International Prostate Symptom Score, prostate size ≤50 or >50 mL, catheterization for ≤3 or >3 days, drained volume at catheterization <1000 or ≥1000 mL, α_1_-blockade before TWOC). The calculations were performed using the Statistical Analysis System (version 9.1.3; SAS Institute Inc. Cary, NC, USA).

## RESULTS

**FIG. 1:**
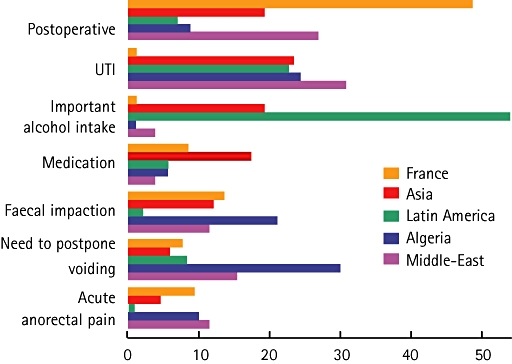
Triggering event in 1785 men catheterized for a precipitated acute urinary retention. UTI, urinary tract infection.

Of the 6074 men enrolled, 4289 (70.6%) had a spontaneous AUR and 1785 (29.4%) had a precipitated AUR. Patient clinical characteristics are provided globally and by region in Table 1. Algeria showed the lowest rate of precipitated AUR (11.9%) whereas Latin America showed the highest rate (43.8%), mainly consecutive to excessive alcohol intake (Fig. 1). Another important triggering event for AUR was surgery with loco-regional/general anaesthesia, especially in France. A previous AUR episode was reported by 16.5% of men, ranging from 10% in France to 24% in the Middle East, within a median delay of 7 months before enrolment. Compared with spontaneous AURs, precipitated AURs were characterized by significantly less severe LUTS (22.3% vs 31.7%, *P* < 0.001), a lower percentage of prostate glands >50 g (36.9% vs 45.4%, *P* < 0.001) and a higher rate of BPH revealed by AUR (44.2% vs 32.9%, *P* < 0.001).

**TABLE 1:** Clinical characteristics of 6074 men catheterized for painful acute urinary retention

	Total (*N* = 6074)	France (*N* = 2618)	Asia (*N* = 1727)	Latin America (*N* = 883)	Algeria (*N* = 755)	Middle East (*N* = 91)
Age, years						
Median	70	72	70	68	72	65
<65, %	27.4	26.0	26.2	37.0	20.5	49.4
65–74, %	39.3	37.6	41.6	37.6	42.1	38.5
≥75, %	33.3	36.4	32.2	25.4	37.4	12.1
Type of AUR, %						
Spontaneous	70.6	71.6	68.8	56.2	88.1	71.4
Precipitated	29.4	28.4	31.2	43.8	11.9	28.6
BPH revealed by AUR, %						
Yes	36.2	33.2	37.6	39.5	40.1	30.8
No	63.8	66.8	62.4	60.5	59.9	69.2
Severity of LUTS*, %						
Mild	13.3	16.8	7.6	14.8	13.6	14.3
Moderate	57.5	61.3	51.8	57.2	56.9	75.0
Severe	29.2	21.9	40.6	27.9	29.6	10.7
DRE estimated prostate size†, g						
<30, %	6.5	6.1	11.3	3.7	0.3	3.3
30–50, %	50.4	44.6	60.3	50.2	43.3	36.3
>50, %	43.1	49.3	28.4	46.1	56.4	60.4
History of previous AUR						
Yes, %	16.5	10.2	23.6	19.6	18.0	24.2
Median delay, months	6.9	7.2	7.5	7.9	4.5	8.4

* Available in 3874 men. Lower urinary tract symptom severity evaluated according to International Prostate Symptom Score.

† Available in 5013 men. AUR, acute urinary retention; BPH, benign prostatic hyperplasia; DRE, digital rectal examination; LUTS, lower urinary tract symptoms.

Catheterization was performed almost exclusively by urologists in France (81.3%) but it was performed by an emergency room physician in up to 40.9% of patients in other regions (Table 2). A urethral catheter was inserted in most cases (89.8%), whatever the region. France showed the highest rate of suprapubic catheter insertion (16.7%). The hospitalization rate for AUR varied between regions, ranging from 1.7% in Algeria to 100% in France.

**TABLE 2:** Initial management of acute urinary retention (AUR) in 6074 men catheterized for painful AUR

	Total (*N* = 6074)	France (*N* = 2618)	Asia (*N* = 1727)	Latin America (*N* = 883)	Algeria (*N* = 755)	Middle East (*N* = 91)
Type of practice, %						
Public	37.4	33.1	85.7	16.9	83.3	40.0
Private	41.9	54.9	1.0	22.3	16.7	10.0
Both	20.7	12.0	13.3	60.8	0	50.0
Type of catheter, %						
Urethral	89.8	82.7	94.5	98.1	93.1	95.6
Suprapubic	8.2	16.7	1.8	1.0	2.3	3.3
In and out	1.8	0	3.7	0.9	4.6	1.1
Unspecified	0.3	0.6	0	0	0	0
Catheterization performed by, %						
Urologist	60.5	81.3	36.5	59.0	50.1	63.7
Emergency room physician	21.1	0.4	35.8	30.6	40.9	33.0
Nurse	15.3	17.3	21.5	8.4	4.4	1.1
Other	3.2	1.0	6.1	2.0	4.6	2.2
Drained volume, %						
<1000 mL	68.4	63.2	72.6	67.7	77.0	75.8
≥1000 mL	31.6	36.8	27.4	32.3	23.0	24.2
Hospitalization for AUR						
Yes, %	57.4	100.0	40.4	12.6	1.7	52.7
Management after catheterization						
TWOC	76.8	72.8	75.4	75.8	93.6	91.2
Immediate surgery	7.2	5.7	12.9	6.5	0.5	4.4
Prolonged catheter and elective surgery	13.3	17.9	9.4	15.1	5.0	4.4
Stent	0.2	0.4	0.1	0	0	0
Indwelling catheter	1.6	1.1	2.4	2.6	0.5	0
Unspecified	1.1	2.1	0	0.2	0.3	0

TWOC, trial without catheter.

After initial catheterization, TWOC was the standard whatever the region (76.8%) (Table 2), with slightly higher rates for precipitated AURs compared with spontaneous AURs (86.1% vs 73% respectively, *P* < 0.001). Overall, 13.3% of men were managed by prolonged catheterization followed by programmed surgery, the highest rates being reported in France (17.9%) and Latin America (15.1%). Immediate surgery was less common (7.2%), the highest rate being reported in Asia (12.9%) and the lowest in Algeria (0.5%). Compared with precipitated AURs, spontaneous AURs were more likely to be treated by surgery, either immediately (8.4% vs 4.3%, *P* < 0.001) or after prolonged catheterization (15.6% vs 7.6%, *P* < 0.001).

**FIG. 2:**
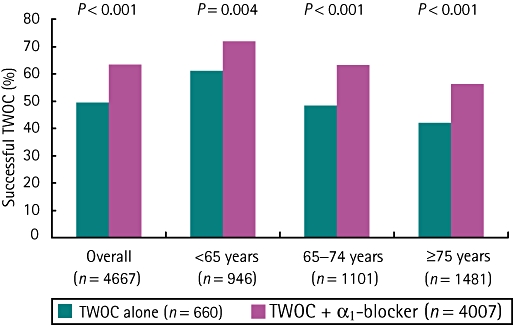
Impact of α_1_-blockade on trial without catheter (TWOC) success rate by age.

In all, 4667 men underwent a first TWOC with an overall success rate of 61.4% (precipitated AUR 66.3%, spontaneous AUR 59.0%, *P* < 0.001). Median duration of catheterization before TWOC ranged from 3 days in France to 8 days in Algeria. Most men (85.9%) received an α_1_-blocker before the TWOC (alfuzosin 68.2%, tamsulosin 16.5%, doxazosin 6.1%, terazosin 2.6%, prazosin 0.1%). Use of an α_1_-blocker before TWOC significantly increased overall the success rate (63.4% vs 49.5%, *P* < 0.001), including for spontaneous (61.4% vs 44.5%, *P* < 0.001) and precipitated (67.5% vs 59.0%, *P* = 0.01) AURs. Similarly, the benefit of α_1_-blockade before TWOC was confirmed irrespective of age (Fig. 2).

**FIG. 3:**
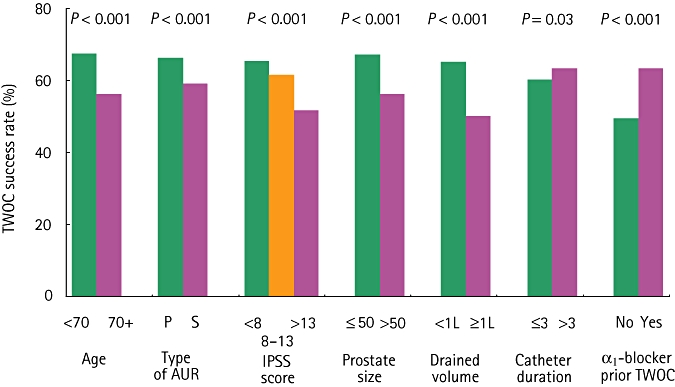
Significant predictors of trial without catheter (TWOC) success in univariate analysis. P, precipitated acute urinary retention (AUR); S, spontaneous AUR; IPSS, International Prostate Symptom Score; catheter duration is expressed in days.

Univariate regression analysis showed that older age (≥70 years), enlarged prostates (>50 g), severe LUTS, large drained volume at catheterization (≥1000 mL) and AUR of spontaneous origin were associated with significantly higher rates of TWOC failure whereas catheterization for >3 days and α_1_-blockade before TWOC increased success rates (Fig. 3). Multivariate analysis with a stepwise procedure confirmed the predictive value of these variables on TWOC outcome except for catheterization duration >3 days, which was no more significant. Allowing for all these factors, α_1_-blocker before a TWOC still doubled the chances of successful TWOC (odds ratio 1.92, 95% CI 1.52–2.42, *P* < 0.001) (Table 3).

**TABLE 3:** Predictors of trial without catheter success rate (multiple logistic regression analysis)

	Odds ratio (95% CI)	*P* value
Age (median)		
<70 years	1	<0.001
≥70 years	0.73 (0.62–0.86)	
Type of AUR		
Precipitated	1	<0.001
Spontaneous	0.70 (0.58–0.70)	
Amount of drained volume		
<1000 mL	1	<0.001
≥1000 mL	0.62 (0.51–0.74)	
α_1_-blocker before TWOC		
No	1	<0.001
Yes	1.92 (1.52–2.42)	
LUTS severity before AUR		
Mild	1	0.51
Moderate	0.93 (0.73–1.17)	<0.001
Severe	0.61 (0.47–0.80)	
Prostate volume		
≤50 g	1	<0.001
>50 g	0.63 (0.53–0.74)	

AUR, acute urinary retention; 95% CI, 95% confidence interval; LUTS, lower tract urinary symptoms; TWOC, trial without catheter.

Of the 2862 men with successful first TWOC, most (86.7%) continued on α_1_-blockade (alone 76.1% or combined either with a 5α-reductase inhibitor, 8.4%, or a plant extract, 2.2%) while being followed up regularly (Table 4). In all, 5.7% had programmed surgery whatever the outcome (spontaneous AUR 6.0%, precipitated AUR 5.2%, *P* = ns) whereas 19.7% would have surgery performed if needed (precipitated AUR 13.0%, spontaneous AUR 23.3%, *P* < 0.001).

**TABLE 4:** Management in case of TWOC

	Total (*N* = 4667)	France (*N* = 1906)	Asia (*N* = 1302)	Latin America (*N* = 669)	Algeria (*N* = 707)	Middle East (*N* = 83)
Duration of catheterization before a first TWOC, days						
Median	5	3	6	6	8	7
≤3 days, %	41.3	65.2	30.8	24.6	19.5	20.7
>3 days, %	58.7	34.8	69.2	75.4	80.5	79.3
α_1_-blockade before TWOC*						
Yes, %	85.9	79.0	89.5	85.1	97.7	92.8
First TWOC (*n*)						
Success rate, %	61.4	50.2	66.0	75.2	69.0	71.1
Outcome if first TWOC is a success†						
Medical treatment, %	88.8	76.8	94.8	89.3	99.6	98.3
Surgery whatever the outcome, %	5.7	8.5	3.1	9.3	1.4	1.7
Surveillance and surgery if needed, %	19.7	24.8	4.5	5.0	53.1	5.1
Other, %	3.4	0.4	0.7	0	0	0
Outcome if first TWOC fails						
Recatheterize and try a second TWOC	43.5	33.4	42.8	45.8	85.8	54.2
Recatheterize and plan surgery	49.0	57.5	48.9	51.2	12.8	33.3
Stent	0.9	1.5	0	0	0	12.5
Long-term catheter	2.8	1.1	7.2	3.0	1.8	0
Other	3.5	6.1	1.1	0	0	0
Second TWOC (*n*)	(782)	(316)	(189)	(76)	(188)	(13)
Success rate, %	29.5	25.9	44.1	30.7	19.7	38.5
Outcome if second TWOC is a success†						
Medical treatment, %	88.8	77.2	97.6	82.6	100	100
Surgery whatever the outcome, %	5.7	7.6	3.1	9.3	1.4	1.7
Surveillance and surgery if needed, %	19.7	24.8	4.5	5.0	53.1	5.1
Other, %	3.4	0.4	0.7	0	0	0
Outcome if second TWOC fails						
Recatheterize and try a third TWOC	18.8	6.2	39.0	26.9	21.2	12.5
Recatheterize and plan surgery	70.7	77.0	51.4	67.3	74.8	87.5
Stent	1.7	4.0	0	0	0	0
Long-term catheter	7.0	8.0	10.5	5.8	4.0	0
Other	3.3	6.2	2.9	1.9	0	0
Third TWOC (*n*)	(102)	(14)	(41)	(14)	(32)	(1)
Success rate, %	26.4	NR	32.5	21.4	18.8	100

* Alfuzosin 68.2%, tamsulosin 16.5%, doxazosin 6.1%, terazosin 2.6%, prazosin 0.1%.

† Sum is higher than 100% because several options may be ticked.NR, not reported, TWOC, trial without catheter.

**FIG. 4:**
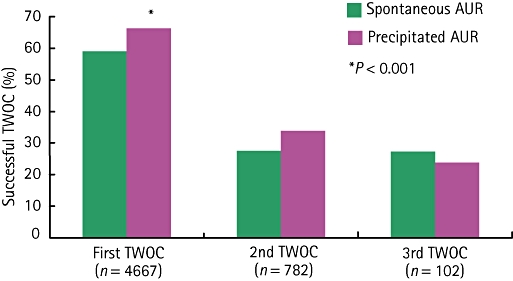
Trial without catheter (TWOC) success rate by type of acute urinary retention (AUR) after one, two and three attempts.

Of the 1798 men who failed a first TWOC, most (49%) were recatheterized and underwent BPH-related surgery whereas 43.5% tried another TWOC after a median of 8 days after the catheterization. The second TWOC was successful in 29.5% of cases (precipitated AUR 34.0%, spontaneous AUR 27.5%, *P* = 0.07), ranging from 19.7% in Algeria to 44.1% in Asia (Table 4; Fig. 4). Of the 542 men who failed a second TWOC, 70.7% were recatheterized and had surgery, 18.8% tried a third TWOC with an overall success rate of 26.4% and 10.5% had another approach.

Catheterization for more than 3 days was associated with a significantly higher rate of adverse events compared with those catheterized for 3 days or less (33.8% vs 19.7%, *P* < 0.001) (Table 5). In patients who were hospitalized, prolonged catheterization also resulted in a slightly higher rate of prolonged hospitalization for adverse events (5.2% vs 3.5%, *P* = 0.007).

**TABLE 5:** Impact of catheter duration of the adverse event profile

	≤3 days (*n* = 1853), *n* (%)	>3 days (*n* = 2638), *n* (%)	*P* value
At least one adverse event during catheter period	281 (19.7)	1453 (33.8)	<0.001
Haematuria	177 (9.6)	274 (10.4)	0.35
Asymptomatic bacteriuria	81 (5.7)	579 (13.5)	<0.001
Lower urinary tract infection	49 (3.4)	308 (7.2)	<0.001
Urosepsis	9 (0.6)	51 (1.2)	0.06
Urine leak	52 (3.7)	297 (6.9)	<0.001
Catheter obstruction	12 (0.8)	133 (3.1)	<0.001
Other adverse events	14 (1.0)	72 (1.7)	0.05
Prolongation of hospitalization for adverse event	47 (3.5)	209 (5.2)	0.007

## DISCUSSION

Acute urinary retention represents the commonest indication for BPH-related surgery [3] but its management is still not standardized because of a lack of existing guidelines. The cross-sectional survey described here evaluates prospectively the management of AUR in a wide range of healthcare systems. The key findings may be summarized as follows. First, initial management of AUR mainly consists of urethral catheterization followed by TWOC but important differences exist between countries regarding hospitalization rate, duration of catheterization and management of TWOC outcome. Second, it confirms in real-life practice that older age, severe LUTS, large drained volume at catheterization and AUR of spontaneous origin favour TWOC failure. Nevertheless, considering all these factors, α_1_-blockade before a TWOC still doubles the chances of success. Third, prolonged catheterization (>3 days) does not influence TWOC outcome in multivariate analysis but is associated with a greater comorbidity and a prolonged hospitalization rate because of adverse events. Fourth, if the first TWOC fails, another TWOC may be attempted with a success rate of 29.5%. Last, only a few men (one in four) undergoes BPH-related surgery after a first episode of AUR, elective surgery being preferred to immediate surgery.

Before discussing the policy implications of the survey, some methodological aspects should be considered. There was no random selection of participating centres, which raises the question if they were fully representative of standard of care in a given country. Our survey is the largest ever conducted in this setting and was specifically designed to include at least 50% of urology centres in each country, both in public and private practice. In our opinion, it provides the physician with reliable and useful information on AUR management in a given country. It also allows comparison of daily practices to a wide range of healthcare systems, showing not only common trends between countries but also important differences, which need to be addressed to optimize patient care.

In this survey, the hospitalization rate for AUR varied between countries, ranging from 1.7% in Algeria to 100% in France. This obviously reflects major differences between healthcare systems but it also questions the necessity of hospitalizing men presenting with AUR. In a UK survey, 65.5% of physicians automatically admitted men catheterized for AUR whereas 19.3% would only admit those with renal impairment [10]. According to the UK National Prostatectomy Audit, men discharged home after catheterization to await surgery showed a higher rate of uncomplicated urinary tract infection and consequently received more antibiotics than those kept in hospital but there was no increased risk of major infective complications [15,16]. Nevertheless, cost savings of sending the patient home should be balanced against patient discomfort and anxiety generated by the catheter being *in situ* outside the hospital.

The survey also establishes TWOC as standard practice worldwide (76.8%) in men catheterized for AUR, with an overall success rate of 61%. The advantage of TWOC is that it allows BPH-related surgery to be performed later, and without the morbidity associated with a catheter. According to the UK National Prostatectomy Audit, deferred surgery for AUR is associated with significantly reduced risks of perioperative complications and postoperative death at 30 days [15]. This ‘window of opportunity’ possibly allows patients, who are usually elderly with comorbid conditions, to face surgery in ‘fitter condition’ with a better detrusor function. Our results are in agreement with those of a UK survey conducted among 410 consultant urologists [10] showing that most of them (73.9%) were using TWOC, mainly after 2 days catheterization (48.5%).

The large database collected here (4667 men with TWOC), allowed us to conduct a robust analysis of predictors of TWOC outcome. Multivariate analysis identified older age (≥70 years), large prostates (≥50 g), severe LUTS, large drained volume at catheterization (≥1000 mL) and AUR of spontaneous type as predictors of TWOC failure. Nevertheless, even in the presence of these adverse factors, α_1_-blockade before TWOC almost doubled the chances of success. This is in agreement with the ALFAUR (Alfuzosin in Acute Urinary Retention) study, which also reported a higher rate of failed TWOC in men age ≥65 years or with a drained volume ≥1000 mL and a twice as high chance of success in men treated with alfuzosin within the 2–3 days before TWOC [11]. The benefit of α_1_-blockade before TWOC was further established by a Cochrane metaanalysis of five randomized trials, including four with alfuzosin [12]. It confirms an old concept, which was initially suggested by Caine *et al*. in 1976 [17], that AUR is the consequence of a sudden sympathetic stimulation causing an acute increase in smooth muscle tone in the lower urinary tract. By relaxing the smooth muscle tone, α_1_-blockers would then favour a return to normal voiding.

It has been reported that chances of successful TWOC increase with the duration of catheterization [18]. Our survey also showed in a univariate analysis that catheterization for >3 days was associated with higher TWOC success rates. Nevertheless, in multivariate analysis, duration of catheterization before TWOC was no more significant. In our opinion, considering the fact that longer catheter duration significantly increases the risk of complications such as urinary tract infections, urine leak and catheter obstruction, all efforts should be made to try to minimize the duration of catheterization and so reduce comorbidity and healthcare costs.

When the first TWOC was successful, 5.7% of patients had deferred surgery irrespective of the outcome whereas 19.7% would undergo surgery only if needed. Although figures appear slightly higher for spontaneous AURs compared with precipitated AURs, these results show that a first episode of AUR is not an absolute indication for surgery and that deferred surgery ‘if needed’ is the preferred option among urologists worldwide. In the second phase of the ALFAUR study, which randomized patients with successful TWOC to alfuzosin 10 mg once daily or placebo for 6 months, BPH-related surgery was needed in 17.1% of patients treated with alfuzosin vs 24.1% of patients receiving placebo, mainly because of AUR relapse [19]. A retrospective analysis of 165 527 men admitted for AUR between 1998 and 2003 in NHS hospitals revealed that BPH-related surgery after a first episode of AUR tended to decrease over time, being required in 32% of men with spontaneous AUR and 5.8% of men with precipitated AUR in 2003 [13]. Conversely, the percentage of patients readmitted for AUR relapse slightly increased over the same period. These data emphasize the need to evaluate carefully the risk of unfavourable outcome after TWOC to identify patients who may benefit from a prolonged medical therapy without developing complications and those who need to undergo surgery in the short-term. In our survey, enlarged prostates, severe LUTS, large drained volume and AUR of spontaneous origin were associated with an increased risk of TWOC failure. These variables have also been identified as predictors of recurrent AUR/surgery after a successful TWOC [9,18,20]. They could be used to select patients who should rapidly be offered a surgical procedure.

In conclusion, this large cross-sectional survey, which enrolled 6074 men with BPH catheterized for AUR, evaluates prospectively the management of this urological emergency in a wide range of healthcare systems. It shows that urethral catheterization followed by a TWOC is the standard practice worldwide and that α_1_-blockade before TWOC doubles the chances of success. Nevertheless, it also highlights important differences between countries regarding hospitalization rate, duration of catheterization and management of TWOC outcome, which are attributed to lack of guidelines for AUR management. Multivariate analysis of predictors of TWOC outcome reveal that catheterization for >3 days does not influence the success rate but is associated with a greater comorbidity and prolonged hospitalization for adverse events. Older age, severe LUTS, large drained volume at catheterization and AUR of spontaneous origin favour TWOC failure. As these variables also predict the risk of recurrent AUR/surgery after a successful TWOC, they could be used to identify the subgroup of patients that cannot be managed by medical therapy alone and should rapidly undergo surgery.

## Abbreviations

AUR: acute urinary retention
LUTS: lower urinary tract symptoms
PVR: postvoid residual urinary volume
TWOC: trial without catheter.
